# Gait Variability as a Potential Motor Marker of Cerebellar Disease—Relationship between Variability of Stride, Arm Swing and Trunk Movements, and Walking Speed

**DOI:** 10.3390/s24113476

**Published:** 2024-05-28

**Authors:** Daniel Kroneberg, Astrid Nümann, Martina Minnerop, Maria Rönnefarth, Matthias Endres, Andrea A. Kühn, Friedemann Paul, Sarah Doss, Susanne Solbrig, Morad Elshehabi, Walter Maetzler, Tanja Schmitz-Hübsch

**Affiliations:** 1 Department of Neurology with Experimental Neurology, Charité–Universitätsmedizin Berlin, corporate Member of Freie Universität Berlin and Humboldt-Universität zu Berlin, Charitéplatz 1, 10117 Berlin, Germany; 2 Berlin Institute of Health, Charité–Universitätsmedizin Berlin, Charitéplatz 1, 10117 Berlin, Germany; 3 Experimental and Clinical Research Center, a cooperation of Max-Delbrueck Center of Molecular Medicine and Charité–Universitätsmedizin Berlin, Lindenberger Weg 80, 13125 Berlin, Germany; tanja.schmitz-huebsch@charite.de; 4 Institute of Neuroscience and Medicine (INM-1), Research Center Jülich, 52425 Jülich, Germany; 5 Institute of Clinical Neuroscience and Medical Psychology, Medical Faculty & University Hospital Düsseldorf, Heinrich Heine University Düsseldorf, 40225 Düsseldorf, Germany; 6 Center for Movement Disorders and Neuromodulation, Department of Neurology, Medical Faculty & University Hospital Düsseldorf, Heinrich-Heine University Düsseldorf, 40225 Düsseldorf, Germany; 7 Center for Stroke Research Berlin, Charitéplatz 1, 10117 Berlin, Germany; 8 NeuroCure Cluster of Excellence, Charité–University Medicine Berlin, Charitéplatz 1, 10117 Berlin, Germany; 9 German Center for Neurodegenerative Diseases (DZNE), Partner Site Berlin, Charitéplatz 1, 10117 Berlin, Germany; 10 German Centre for Cardiovascular Research (DZHK), Partner Site Berlin, 10117 Berlin, Germany; 11 German Center for Mental Health (DZPG), Partner Site Berlin, 10117 Berlin, Germany; 12 NCRC-Neuroscience Clinical Research Center, Charité–Universitätsmedizin Berlin, corporate Member of Freie Universität Berlin and Humboldt-Universität zu Berlin, Charitéplatz 1, 10117 Berlin, Germany; 13 Department of Neurological Sciences, University of Nebraska Medical Center, Omaha, NE 68198, USA; 14 Department of Neurodegenerative Diseases, Center for Neurology, Hertie Institute, Hoppe-Seyler-Straße 3, 72076 Tübingen, Germany; 15 Department of Neurology, Universitätsklinikum Schleswig-Holstein, Arnold-Heller-Straße 3, 24105 Kiel, Germany

**Keywords:** gait assessment, cerebellar ataxia, gait and posture, motor performance marker

## Abstract

Excessive stride variability is a characteristic feature of cerebellar ataxias, even in pre-ataxic or prodromal disease stages. This study explores the relation of variability of arm swing and trunk deflection in relationship to stride length and gait speed in previously described cohorts of cerebellar disease and healthy elderly: we examined 10 patients with spinocerebellar ataxia type 14 (SCA), 12 patients with essential tremor (ET), and 67 healthy elderly (HE). Using inertial sensors, recordings of gait performance were conducted at different subjective walking speeds to delineate gait parameters and respective coefficients of variability (CoV). Comparisons across cohorts and walking speed categories revealed slower stride velocities in SCA and ET patients compared to HE, which was paralleled by reduced arm swing range of motion (RoM), peak velocity, and increased CoV of stride length, while no group differences were found for trunk deflections and their variability. Larger arm swing RoM, peak velocity, and stride length were predicted by higher gait velocity in all cohorts. Lower gait velocity predicted higher CoV values of trunk sagittal and horizontal deflections, as well as arm swing and stride length in ET and SCA patients, but not in HE. These findings highlight the role of arm movements in ataxic gait and the impact of gait velocity on variability, which are essential for defining disease manifestation and disease-related changes in longitudinal observations.

## 1. Introduction

Gait ataxia is a clinical feature of cerebellar pathology and often the presenting symptom of neurodegenerative ataxias [1], which may progress to inability to walk in later disease stages [2]. Clinically, ataxic gait is defined as slowed with a broadened base, as well as an instable stepping pattern and irregular movement path. In early stages, its distinction may need more challenging tasks, such as tandem gait [3]. Instrumental gait assessment has been applied to delineate the kinematic features of ataxic gait [4,5] and their quantification has been proposed as digital motor markers for disease monitoring [6]. Group differences in spatial and temporal gait features between ataxic and healthy subjects showed some inconsistency among previous reports with different etiology, different testing protocols, or different methods of assessment as possible explanations [7,8,9]. In contrast, excessive spatial and temporal variability of stepping has been consistently reported as a finding in ataxias of different etiologies and may have predictive value for future falls [4,7,10,11]. Most importantly, increased gait variability and postural instability have been shown to precede the manifestation of gait ataxia in various hereditary ataxias [3,7,12,13,14,15]. Thus, if reliably quantified, such performance-based outcome assessments may contribute to the definition of prodromal disease stages [16]. Regarding perspectives for their application in disease monitoring, a recent longitudinal observation in SCA type 3 reported changes for two quantitative gait measures—the variability of stride length and lateral body sway—whereas changes in the clinical rating did not reach significance at a 1-year follow up [14].

Current descriptions of gait domains focus on features of locomotor stepping [17]. Far less is known about the characteristics of trunk and arm movements in healthy and diseased states, with evidence of their contribution to dynamic gait stability [18] despite a lack of clear presumptions on their control during gait. Second, prior evidence on the speed-dependency of gait features has been incorporated into consensus testing protocols using different speed instructions [6]. The lowest variability of stride time is generally seen around gait velocities of subjectively preferred or comfortable speed [19]. In ataxias, in addition to increased overall variability, slowed gait speed is commonly observed. Furthermore, the speed dependency of stride variability has been reported to differ between conditions, for example, between cerebellar ataxia and bilateral vestibular failure [20,21].

We here provide a comprehensive description of the trunk and arm movements during gait and their relationship with gait velocity in cohorts with manifest or suspected cerebellar pathology, as well as a group of healthy elderly. Stride length and its spatial variability were also included in this report to contextualize our main findings.

The sample was pooled from three single-center investigations performed at different sites, which all used the same inertial sensor system for recording. For this analysis, identical procedures for postprocessing and quality control were applied to all data. The investigation included patients with spinocerebellar ataxia type 14, which has been more recently termed SCA-PRKCG (protein kinase C gamma). Our SCA cohort is a subset of a multicenter cohort of SCA-PRKCG published previously [22]. In this autosomal-dominant ataxia with selective loss of Purkinje cells, the phenotype, as well as MR imaging results, are consistent with pure cerebellar involvement [23,24,25]. This group can thus be considered as representative for ataxic gait. We further included data from patients with essential tremor (ET), who often feature (mildly) ataxic gait in the advanced stages of disease [26] or other cerebellar signs such as intentional tremor [27]. The data of healthy elderly subjects represent a subset of the TREND study, which is a longitudinal observational study that included unselected persons aged 55 or older to investigate the evolution of neurodegenerative disorders with aging [28]. These data are included to allow preliminary inferences on the physiology of trunk and arm movement control.

Descriptive results per group and speed category may serve as useful reference for plausibility checks in future investigations. While no strong hypothesis on the speed dependency of arm and trunk features has been put forward, we assumed that both patient groups would show differences from the physiological patterns observed in healthy elderly. Furthermore, we hypothesized that the performance in the ET group would be qualitatively similar to the SCA group though less pronounced. If arm movements during gait are subject to the same control mechanisms as leg movements, their relation to gait speed, as well as the effects of ataxia, should be expected to be similar. Alternatively, arm movement—as well as truncal movement—may be affected differently by cerebellar pathology and thus may even compensate for gait ataxia.

This study aims to provide a quantitative description of arm and trunk movements and their variability, along with established biomarkers of lower limb movements at different gait velocities that may be useful both as references during diagnostic procedures, as well as during the longitudinal monitoring of disease severity or the progression of the clinical correlates of cerebellar pathology.

## 2. Methods

### 2.1. Study Cohorts and Dataset

Clinical and kinematic data were available from 10 patients (5 female) with clinically manifest and genetically confirmed neurodegenerative spinocerebellar ataxia type 14 (SCA-PRKCG, formerly SCA14), which is a subset of a prospective observational study in this is disorder [22]. Patients had an average age of 54 ± 14 years [range 27–69], an average BMI of 29 ± 5 kg/m^2^ [range 22–40], and an average symptom severity score of 10.7 ± 9.5 [range 3–20.5] of maximum possible 40 points as assessed by the scale for the assessment and rating of ataxia (SARA) [29]. Of note, the sample included one incipient case with SARA rating of 3 who scored 0 in SARA item 1 (gait). Spatiotemporal characteristics of stride length and velocity have previously been reported for 8 patients from this cohort [30].

For ET patients, clinical and kinematic data were available from 12 patients (6 female) with average age of 67 ± 10 [range 48–82] years and BMI of 25 ± 2 kg/m^2^ [range 21–28]. On average, this group featured mild tremor (TRS of 19 ± 10 (of maximum 116, range 7–38)), as well as mild cerebellar signs (SARA score 6.4 ± 3.2, [range 2–11]). Stride variability for this cohort has been reported previously [31]. These patients were under evaluation for surgical tremor therapy and did not require walking aids, nor had they concurrent conditions affecting gait performance (i.e., neuropathy, vestibular disorders, musculoskeletal impairments).

We further used data from 67 healthy elderly from the TREND cohort (27 female, average age 69 ± 5 years [range 58–86], BMI not available) who were assessed during the third visit (2013/14) of the TREND study [32] and had been included in a previous investigation of stride variability [31]. TREND only included subjects without functionally relevant disturbances of gait or balance. For this analysis, we selected subjects with recordings available from both comfortable and fast walking speed categories.

All studies were approved by the respective institutional review boards (Charité Universitätsmedizin Berlin EA1/267/12, EA2/015/16 and the Medical Faculty of the University of Tübingen Nr. 90/2009BO2). All subjects provided informed consent.

### 2.2. Protocol for Gait Assessment

Gait assessment for all cohorts was performed with the Mobility Lab^TM^ system (Mobility Lab V1 hardware, APDM, Portland, OR, USA), consisting of 6 body-worn inertial sensors (Opal) attached to wrists, ankles, and over the sternum and lower back according to manual. Walking performance for SCA and ET groups was assessed over 10 m, which subjects walked twice at three different subjective speeds in consistent order (comfortable, slow, fast), starting from standing position. In the TREND study, HE walked 20 m bouts marked with pylons back and forth for 1 min without specific instructions for turning. Raw data were sampled at 128 Hz, processed within the Mobility Lab software V1.0.0.20150330213 and exports of stridewise timecoded values of gait parameters per trial, and trial averages and coefficient of variability (CoV) were generated, thus defined as the standard deviation divided by the mean. Reliability of CoVs of gait parameters over shorter distances used in this study has previously been validated and reported [31]. The manufacturer’s algorithms have previously been validated against other motion analysis technologies by third parties [33] and through numerous studies (https://apdm.com/publications accessed on 8 December 2023). 

Spatial and temporal parameters extracted from Mobility Lab software export—after excision of steps in turns—were stride length [%stature], stride velocity (representing gait speed) [%stature/s], angular range of motion of arms [degrees], peak arm swing velocity [degrees/s], and angular range of motion of trunk in frontal (i.e., mediolateral), horizontal (i.e., rotational), and sagittal plane (i.e., anterior–posterior) [degrees]. Stride length and velocity are reported normalized to individual height, as taller subjects naturally walk faster [34] and with longer strides [35]. To facilitate comparison with other studies, absolute values of stride length and velocity are reported alongside with normalized values in Table 1A.

### 2.3. Statistical Analyses

For all trials, from the exports provided by the manufacturers software, lengths and durations of each recorded stride were plotted against their time stamps and inspected to determine if turns had been detected and excised properly, as described previously [31]. Shapiro–Wilk tests were performed for gait parameters and clinical scores on a group level to determine normality of distributions. Velocities at slow and fast walking were calculated as relative to velocity at comfortable walking speed. Differences of gait parameters at different walking speed categories were investigated with either repeated measure ANOVAs or Friedman tests, as well as respective post hoc paired *t* tests or Wilcoxon tests depending on normality of distribution of each parameter to avoid violations of test assumptions. Between-group comparisons were conducted with Kruskal–Wallis tests for comfortable and fast walking between SCA, ET, and HE and for slow walking with Mann–Whitney U tests between SCA and ET patients. To investigate if absolute walking speed, here named gait velocity, explained the variance of gait parameters and their respective CoV values, trials were pooled within group across all speed conditions, and linear regressions were applied with gait velocity as independent and gait parameter or corresponding CoV as response variable. An alpha level of 0.05 was considered significant. Statistical analyses were conducted with exploratory intent.

## 3. Results

**Figure 1:**
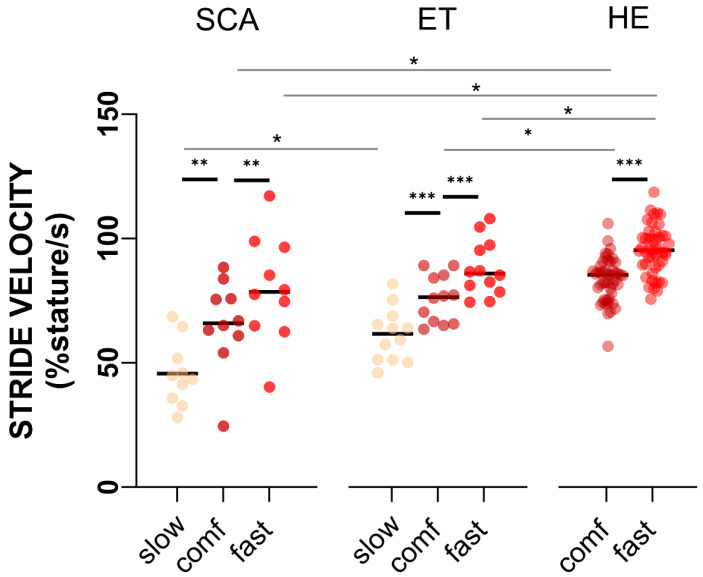
Distribution of stride velocity (%stature/s) for each walking speed category and cohort. * indicates significant differences between walking speed categories within cohorts or across cohorts. * *p* < 0.05, ** *p* < 0.01, *** *p* < 0.0001.

**Figure 2:**
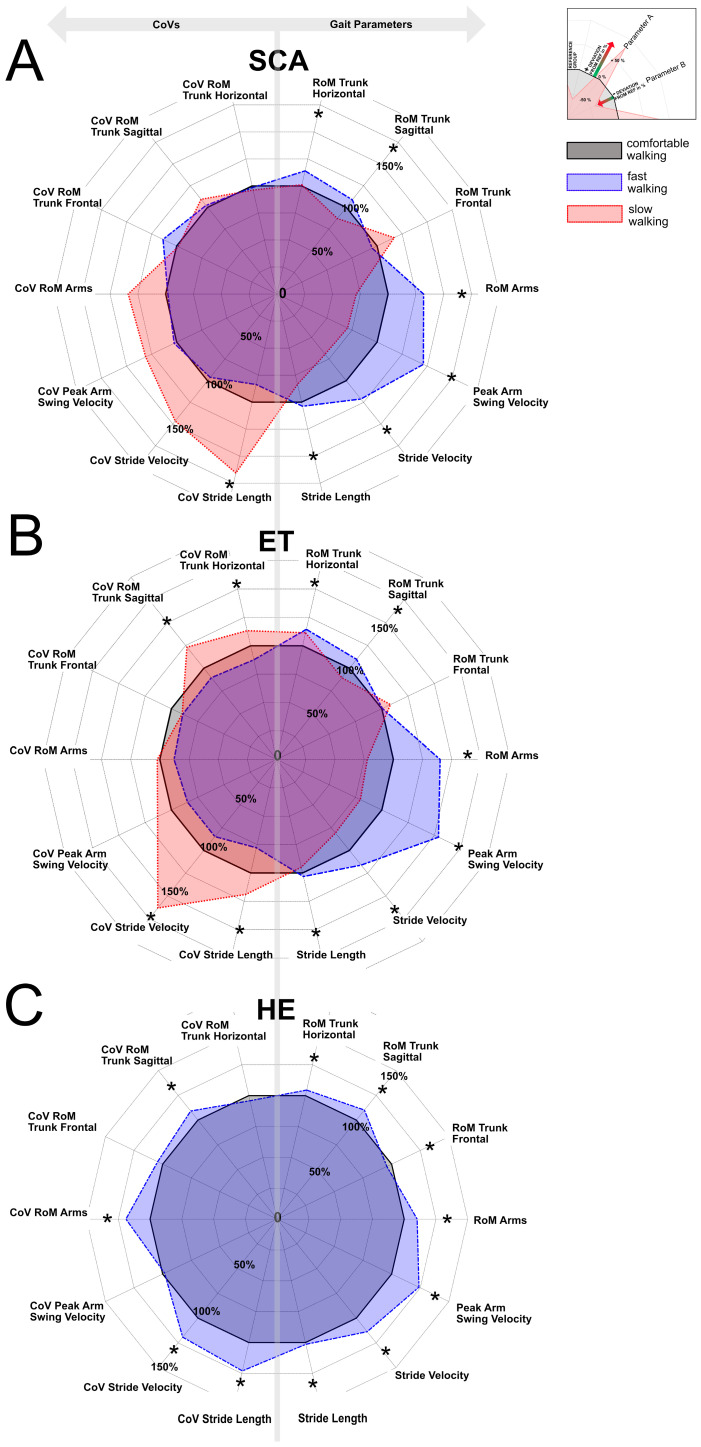
(**A**–**C**) Spatiotemporal gait parameters (right half of plots) and their respective coefficients of variability (CoV) mirrored to the left half of plots. Values of parameters and CoV values for slow and fast walking speed category are normalized to results at comfortable walking speed (black dotted line) to display relative differences. * indicates significant differences of gait parameters between speed categories.

The distributions of the absolute stride (i.e., gait) velocities per walking speed category and group are visualized in Figure 1. The averages of all gait parameters and respective CoVs at different walking speed categories are displayed in Table 1A,B and Figure 2A–C (and supplemental Figure S1).

**Figure 3:**
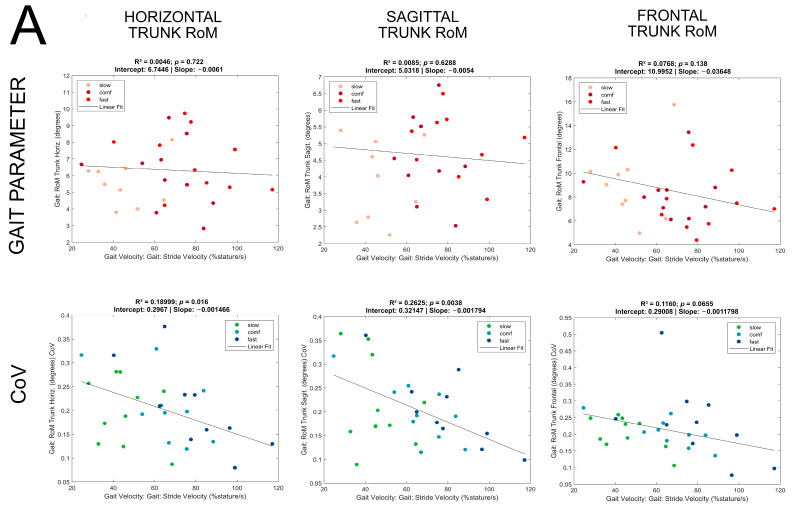

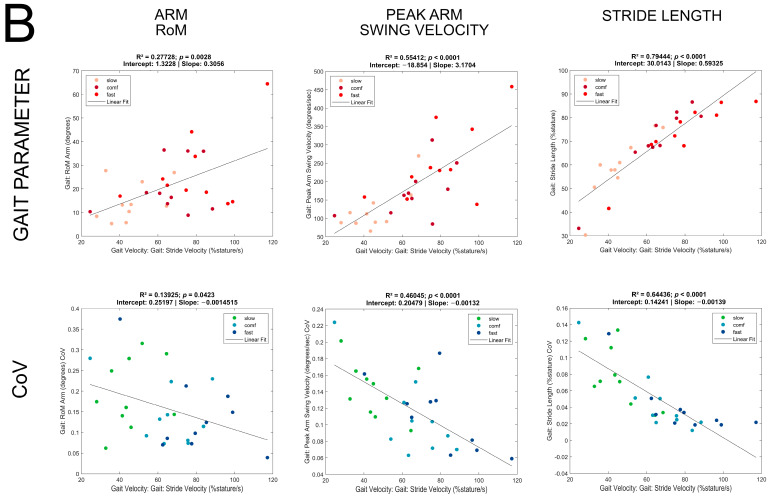
(**A**,**B**) Dependence of gait parameters on velocity in SCA: gait parameters and respective CoV values (y axis) plotted against absolute gait velocity (x axis). Dots represent individual values, with colors indicating the walking speed category in which they were obtained. Linear fit of linear regression modeling is superimposed for each parameter/CoV with results for R^2^, *p*-value, slope, and intercept above each plot.

The slopes and intercepts of significant linear regressions are reported per group in Table 2A,B and illustrated for the SCA group in Figure 3A,B (see Supplemental Figure S3A,B for ET patients and Figure S5A,B for HE).

An average of 14.3 [range 7–23] and 13 [range 6–22] gait cycles per individual and speed category were available in the SCA and ET patients, respectively, while more (47.6 [range 28–74]) gait cycles were available from HE cohort due to differences in testing protocols. Of note, average age was significantly lower for SCA compared to ET (*p* = 0.024) and HE (*p* < 0.0001).

### 3.1. Patients with Cerebellar Ataxia

Average gait velocity for comfortable speed was 65.8% stature/s and reached ratios of 69.4% [51.8–114.0] and 121.1% [102.7–163.6] of the average at comfortable speed in slow and fast categories, respectively.

Faster walking speed increased trunk and arm movements, as well as stride length: Repeated-measure analyses revealed differences between walking speed categories, with more horizontal and sagittal trunk deflections, arm excursion, and peak arm swing velocity, as well as stride length with faster speed. Respective testing of CoV values revealed differences for the CoV values of stride length and a trend for CoV of peak arm swing velocity (*p* = 0.07), with higher CoV in the slow speed category. No differences were shown for trunk movement in the frontal plane.

Arm movements and velocities, but not trunk movements, were predicted by absolute gait velocity: To account for interindividual differences of performance within instructed walking speed categories, gait parameters and respective CoV values were pooled within the groups, irrespective of the walking speed category in which they were recorded. In linear regression models, no dependency on absolute walking velocity could be shown for trunk RoMs in any direction. In contrast, the RoM of arms (R^2^ = 0.28) and the peak arm swing velocity (R^2^ = 0.55) were predicted by the gait velocity, and the strongest prediction was seen for the stride length (R^2^ = 0.79).

Dependence of the CoV values on the absolute gait velocity was lowest for trunk and highest for stride length: Regression models for the CoV values showed some prediction in that a lower absolute gait velocity predicted a higher CoV of the horizontal trunk RoM (R^2^ = 0.19), the CoV of sagittal trunk RoM (R^2^ = 0.26), and CoV of arms RoM (R^2^ = 0.14), but only showed a trend for the CoV of frontal trunk RoM (R^2^ = 0.11, *p* = 0.07). Stronger predictions were seen for the CoV of peak arm swing velocity (R^2^ = 0.46) and CoV of stride length (R^2^ = 0.64).

### 3.2. Patients with Essential Tremor

The average gait velocity at comfortable speed was 75.8% stature/s and reached ratios of 80% [72.5–96.0] and 116.6% [97.1–145.0] of the average at comfortable walking speed in the slow and fast speed walking categories, respectively.

Repeated-measure analyses revealed differences in trunk movements in horizontal and sagittal planes and their respective CoV values differed across the speed categories: the horizontal trunk RoM was lowest in comfortable (5.2°) versus slow (5.8°) versus fast (6°) walking speed category, and the sagittal trunk RoM increased from slow (3.9°) to comfortable walking speed category (4.3°). No such differences were shown for frontal trunk RoM and CoV. The RoM of arms and the peak arm swing velocities, but not their CoV values, increased with faster walking. Stride length increased from slow to comfortable to fast condition (all *p* < 0.01), while CoV decreased from the slow to fast walking speed category.

Similar to the SCA group, the linear regression models were not significant for trunk RoMs in horizontal, sagittal, and frontal planes as dependent variables. RoM of arms (R^2^ = 0.36) and peak arm swing velocity (R^2^ = 0.46) were predicted by gait velocity, and, again, the strongest prediction was seen for stride length (R^2^ = 0.57).

Gait velocity was weakly predictive for CoV of horizontal trunk RoM (R^2^ = 0.18), CoV of the sagittal trunk RoM (R^2^ = 0.17), CoV of the arms RoM (F(1,34) = 5.3; R^2^ = 0.13, *p* = 0.028), and CoV of stride length (F(1,34) = 18.6; R^2^ = 0.33, *p* = 0.0001) but not CoV of the frontal trunk RoM or CoV of the peak arm swing velocity. As in the SCA group, slower gait velocity predicted higher CoV values.

### 3.3. Healthy Elderly

Average gait velocity increased from 84.0% stature/s at comfortable walking to 95.5% stature/s at fast walking, and thus represents 114% [100.1–135.4] of the average at comfortable speed.

When compared across walking speed categories, larger trunk RoM in the horizontal and sagittal planes were observed in fast compared to comfortable speed category, while frontal trunk RoM was smaller. RoM of arms, peak arm swing velocities, and stride length all increased from the comfortable to fast walking speed category. Unlike in SCA and ET cohorts, CoV values of sagittal trunk RoM, arm RoM, and stride length were higher in fast speed category.

Linear regression models were not significant for trunk RoMs in horizontal and frontal planes as dependent variables and explained only a small proportion of the variance in the sagittal trunk RoM (R^2^ = 0.04, *p* = 0.02) and RoM of the arms (R^2^ = 0.08). Peak arm swing velocity (R^2^ = 0.44) and stride length (R^2^ = 0.39) were substantially predicted by gait velocity but none of the CoV values of the parameters investigated.

### 3.4. Group Comparison per Walking Speed Category

For the distribution of stride velocities per walking speed category and group, see Figure 1.

The SCA group was characterized by impaired RoM and velocity of arm swing and impaired stride length and velocity, which was parallelled by increased variability of stride length and velocity at comfortable walking speed. The trunk movements and their variability were not as useful to distinguish between cohorts at any walking speed. Supplementary Figure S6 illustrates the cohort differences per walking speed categories.

Kruskal–Wallis tests and consecutive post hoc Mann–Whitney U tests indicated differences between the cohorts for the following gait parameters: At comfortable walking speed category, arm RoM was smaller for the SCA cohort compared to HE (*p* = 0.009); arm swing velocity was reduced in SCA (*p* = 0.012) and ET (*p* = 0.048) compared to HE. Stride velocity was highest for HE cohort compared to the SCA (*p* = 0.003) and ET (*p* = 0.01). Stride length was shortest for SCA compared to ET (*p* = 0.03) and HE (*p* < 0.0001). CoV values of stride velocity were largest for SCA compared to ET (*p* = 0.01) and HE (*p* < 0.0001), and CoV of stride length was larger in SCA than in HE (*p* = 0.0004), while the difference to the ET group was a trend (*p* = 0.08). No between-group differences were detected for trunk RoM values and their corresponding CoV values in all directions, the CoV of arm RoM, and CoV of peak arm swing velocity.

In the fast walking speed category, stride velocity was largest in HE compared to SCA (*p* = 0.008) and ET (*p* = 0.03), and stride length was shortest for SCA compared to ET (*p* = 0.025) and HE (*p* < 0.0001) groups. CoV of stride velocity was lowest in ET compared to SCA (*p* = 0.009) and HE (*p* = 0.005). CoV of stride length was largest in SCA compared to ET (*p* = 0.0034) and HE (*p* = 0.045). No differences were detected for trunk RoM in all directions, arm RoM, nor arm peak velocity and its respective CoV values.

For slow walking speed, only groups of SCA and ET could be compared. In SCA group, while stride velocity (*p* = 0.009) and stride length (*p* = 0.0002) were reduced compared to ET, the corresponding CoV values were larger for stride velocity and stride length (*p* = 0.001 and *p* < 0.0001). No differences were detected for trunk RoM in all directions, arm RoM, nor the arm peak velocity and its respective CoV values.

## 4. Discussion

This study adds to the limited reports on arm and trunk movements during gait and addresses important issues for the application of gait analysis in various disorders with cerebellar involvement. Using a commercially available sensor system and task-based testing protocol, it provides a comprehensive description of trunk and arm movements and their variability as a reference point for future investigations. Our findings on their speed dependency during gait may help to stimulate further discussions on appropriate normalization procedures. The contrast of groups with manifest SCA and ET to HE highlights shared features in both disorders and adds to previous descriptions of the kinematic features of ataxic gait.

Specifically, trunk deflections in the frontal plane—unlike deflections in the sagittal or horizontal plane—did not increase with higher walking speed but showed a specific peak at the slow speed condition in parallel to peaks of step variability. When groups were compared at the comfortable walking speed category, reduced ranges of motion were similarly seen for stride movements and arm swing in groups with cerebellar disease (SCA and ET) compared to HE, while higher variability was only confirmed for stride movements. These features relate to the independent gait domains of pace and variability as put forward by Lord and colleagues [17], which were here extended to also include arm swing. In addition, there was excessive variability of stride length and stride velocity in the SCA group. Of note, the visualization of the data suggests an increased trunk RoM in the horizontal and sagittal—but not frontal—plane, as well as increased trunk movement CoV values—most pronounced in the frontal plane—as other possibly distinctive features of disorders with cerebellar pathology when compared to HE. This pattern was similar for both disease groups, but it may have evaded detection in statistical testing due to type II errors. Thus, conclusions on trunk directional movements are limited by small group sizes in this comparison but surely encourage further investigation.

As hypothesized, a similar set of features distinguished SCA and ET groups from the HE group, and performance in ET group was expectedly positioned intermediately between SCA and HE. This is well in line with the notion of mild cerebellar dysfunction in ET, which was also indicated by the SARA range in this ET group.

With respect to speed dependency, the first fundamental finding is the large overlap of recorded absolute gait velocities between instructed walking speed categories. All groups were able to increase their speeds to >110% of the comfortable walking speed (114% (HE) to 121% (SCA)) and to deliberately slow their walking speeds to 70% (SCA) and 80% (ET) of comfortable walking speed. However, mean absolute gait velocities showed a shift by one speed category from SCA to ET to HE group such that the mean at the fast speed category in the SCA group equalled the mean at the comfortable speed category in the ET group and the same in turn for the ET and HE groups (see Figure 1). This poses a challenge for the interpretation of group comparisons per category of instructed speed and motivated the approach to analyse speed dependency versus absolute walking velocity first.

In this regression analysis performed per group, a substantial increase at higher gait velocities was predicted for arm and leg movements in all groups, therein most pronounced in the SCA group (R^2^ = 0.79 for stride length) but not for trunk movements in disease groups. Again, the SCA and ET groups shared in parts their pattern of speed dependency: the variability described as CoV was higher at slower gait velocity—except for CoV of trunk frontal range of motion, which showed a trend only in the SCA group. The higher variabilities at the slower end of gait velocities may explain the lack of significant regressions for variability measures in the HE cohort, in which no slow speed condition had been recorded. 

With respect to arm movement, as shown in between-group comparisons, changes with speed categories ran rather in parallel to those seen for stride parameters. The consideration of arm movement descriptors in larger studies may allow for hypotheses on their role in gait pathology due to cerebellar dysfunction. In healthy individuals, physical restriction of arm swing during walking increased gait instability and variability, thus underlining its role in physiological gait [18]. The fact that lower than normal arm swing paralleled shorter than normal stride length and lower than normal stride (i.e., gait) velocity in this study is well compatible with prior evidence of a large passive component of arm swing during locomotion [36]. This indicates that rhythm and velocity of arm swing are secondary to those of stepping. On the other hand, spinal interactions of limb movement control have been described [37,38] and also considered in therapeutic approaches [39].

With respect to trunk excursions, speed dependency was minimal and significant only in the (larger) HE cohort for trunk movements in the sagittal plane, i.e., the direction of locomotion. This invariance with speed, together with a lack of group differences between the HE and patient groups, may be interpreted as trunk excursions not contributing to the acceleration of locomotion. However, conclusions on invariance with cerebellar disease seem immature and need investigation in larger samples. Specifically, trunk frontal RoM seems to be an interesting candidate: the co-occurence of larger mediolateral (frontal) trunk excursion with higher variability of stepping at the slow speed category in both disease groups may be explained in the context of gait kinematics and warrants further study. The mediolateral stabilization of the center of mass against gravity requires active control during locomotion, while in healthy walking, stabilization in the anteroposterior (sagittal) direction is passively conveyed by the biomechanics of locomotor forward stepping [40]. In healthy subjects, step length and timing at preferred speed have been shown to optimize for vertical and sagittal stability of head and trunk at the cost of suboptimal mediolateral stability [41]. Previous evidence showed increased step variability at slower than comfortable walking speed conditions in healthy gait and cerebellar ataxias [42]. The most common interpretation of this finding is the role of cerebellar dysfunction for intrinsic timing deficits [43,44]. However, it is well conceivable that decreased mediolateral axial stability in cerebellar disease, most evident in the slow walking condition, may exert direct effects on step placement and timing to counteract the resultant destabilization. Thus, the (lack of) control of trunk movement in the frontal plane may in part contribute to excessive variability of stepping observed at slow speed in our diseased cohorts. As a limitation, no comparison to healthy subjects could be made for slow speed walks in our study. Further study in larger ataxia cohorts, as well as in appropriately matched healthy controls, will help to elucidate this potential interaction. Specifically, the exploration of directional components of step variability may help to delineate speed- and balance-related variability components [45].

With respect to arm movement, as shown in between-group comparisons, changes with speed categories ran rather in parallel to those seen for stride parameters. This is well compatible with prior evidence of a large passive component of arm swing during locomotion [36]. The findings indicate that the rhythm and velocity of arm swing are secondary to those of stepping. On the other hand, spinal interactions of limb movement control have been described [37,38] and also considered in therapeutic approaches [39]. At present, stride length and its CoV seem to be favorable features to describe ataxic gait due to most pronounced group effects. However, the consideration of arm movement descriptors in larger studies may allow for hypotheses on their role in cerebellar gait pathology, as well as in compensatory strategies.

Recently, the variability of trunk movements measured as standard deviations of lumbar range of motion in transverse, coronal, and sagittal directions has been shown to provide decent discrimination between a large cohort of genetically confirmed SCA 1, 2, 3, or 6 and healthy controls with an AUC > 0.7, which was similar to differences in the gait speed (AUC 0.812) [7]. Of note, a similar comparison for presymptomatic carriers only revealed measures of variability as weakly distinctive to healthy controls, while gait speed, stride time and arm RoM did not differ. Unfortunately, arm swing velocity and stride length were not among the features selected for their analysis. Another study provided similar results for the comparison of younger healthy controls to a group of pre-symptomatic SCA2 patients [12]. Here, average trunk deflection in all directions also differed between groups, while arm movements were not reported. A study using a multicamera setup of RGB-D sensors also reported on increased lateral trunk sway during gait measured at the level of the sternum in the SCA3 compared to the HC group [14], while the trunk sway in other directions or the arm RoM were not reported. Recent results from our group using an RGB-D single-camera setup showed increased mediolateral but not anterior-posterior trunk excursions at the level of the shoulders among ataxias of different etiologies [4], while arm swing was not reported. Scattered evidence points to the necessity to streamline the selection of descriptors for gait ataxia [6]. To conclude from our results, these should include trunk movement in frontal plane, measures of arm swing, and testing at slow and fast speed conditions to allow for inferences on speed dependency in analysis.

The latter poses a major challenge both for the comparison of cohorts and longitudinal observation, as gait velocities notably decline with the progression of ataxia [46]. Reported findings imply that the observed CoV may best be gauged against the individual gait velocity. With respect to feature reduction, consistent speed dependencies in all groups point to feature clusters: gait features related to acceleration, i.e., increasing with higher gait velocities such as trunk sagittal, arm, and stride movements; those increasing at slower gait velocities, such as CoVs (and possibly trunk frontal RoM in the SCA group); and gait features invariable with gait velocity, such as trunk horizontal deflection. The findings suggest frontal trunk RoM as a feature subject to different control mechanisms which are affected by cerebellar disease. Interactions with pathology in stride coordination and slowing of gait warrant further study. Spatiotemporal features of gait, arm movements, and trunk movements and their variability presented here may further be valuable on an individual level to monitor and quantify the progression of disease [14], as well as prompt evaluation and adaption of therapies, particularly for cutting-edge targeted therapies for orphan diseases that cannot be evaluated in larger cohorts [47].

As a clear limitation of this secondary analysis, the disease groups were small in size and featured different age ranges. Furthermore, they were not compared to matched healthy controls, but inferences were made by comparison to a larger set of healthy elderly subjects. Due to differences in testing protocols, no group comparison against the HE could be conducted for the slow walking speed category, which has been proposed as a testing condition with highest sensitivity for ataxic gait features. Eventually, in ET patients, gait disturbances may have been present both due to cerebellar pathology related to ET but also due to age-related neurodegeneration.

The number of steps and gait cycles available for delineating parameters and their variability were larger for HE due to differences in assessment protocols. The minimum number of 10–15 gait cycles necessary to estimate gait variability measures has been reported previously for the ET and HE cohorts also used in this study [31], particularly highlighting confounding factors that occur during longer distances. Using different assessment strategies and technologies, the current literature reports a large range of necessary strides to estimate variability, from 30 steps/15 strides [48] to more than 50 [49] and up to hundreds when investigating the variability of dual-task walking [50], yet dependency on context and specific clinical characteristics of cohorts confine the generalizability of such reports.

Comparability is further constrained by some differences in testing protocols. Issues for comparability may arise even with the application of the same commercial IMU system, such as alternative measurement units (e.g., m/s vs. %stature/s), alternative sensor placement in more recent protocol versions (e.g., foot sensor placed on foot versus lower shank, lumbar, and upper trunk) as well asdifferent software versions for processing.

## 5. Conclusions

Arm movements and their variability have been identified as speed-dependent and possibly useful additional descriptors of gait pathology due to cerebellar disease, along with variability of horizontal and sagittal trunk movements. In summary, the results presented here confirm the value of recording gait at various speed conditions within one session to (1) specifically capture the variability of gait measures in (early stages of) cerebellar disorders, (2) allow the analysis of individual speed dependencies for the proper interpretation of potential motor markers at individual level, and (3) provide additional information that may be specific to the underlying condition or pathology.

## Figures and Tables

**Table 1:** (**A**) Parameters of trunk and arm movements at different gait speed categories given per cohort. (**B**) Coefficients of variability for parameters of trunk and arm movements at different gait speed categories given per cohort.

(A)																
Gait Parameter ± STD	SCA						ET						HE			
	Slow		Comf		Fast		Slow		Comf		Fast		Comf		Fast	
Trunk Horizontal RoM [degrees]		6.12		6.05		6.90		5.83		5.23		5.99		5.20		5.44
	±	2.08	±	1.96	±	1.76	±	2.63	±	2.73	±	2.86	±	2.33	±	2.24
Trunk Sagittal RoM [degrees]		4.14		4.75		5.17		3.85		4.30		4.72		4.22		4.65
	±	1.27	±	1.35	±	1.01	±	1.05	±	1.28	±	1.62	±	1.42	±	1.59
Trunk Frontal RoM [degrees]		9.75		8.33		7.93		7.33		6.78		6.81		8.71		8.34
	±	3.51	±	1.99	±	2.63	±	2.55	±	2.12	±	2.21	±	2.77	±	2.74
Arms RoM [degrees]		14.69		20.59		27.15		20.15		25.94		36.32		31.99		35.24
	±	7.89	±	10.62	±	15.29	±	14.37	±	16.59	±	20.16	±	13.92	±	14.22
Peak Arm Swing Velocity [degrees/s]		122.47		173.59		253.82		149.96		188.37		289.84		233.48		289.18
	±	56.37	±	65.18	±	100.10	±	58.70	±	69.34	±	101.93	±	75.14	±	82.29
Stride Velocity [%stature/s]		45.72		65.83		79.73		61.26		75.80		87.99		83.96		95.46
	±	12.30	±	17.03	±	20.46	±	10.35	±	9.13	±	10.65	±	8.48	±	9.78
Stride Velocity [m/s]		0.76		1.10		1.34		1.01		1.27		1.50		1.43		1.63
	±	0.18	±	0.28	±	0.35	±	0.16	±	0.16	±	0.20	±	0.14	±	0.17
Stride Length		59.18		70.82		73.52		77.93		82.06		84.65		85.90		87.09
[%stature]	±	12.58	±	14.37	±	12.61	±	6.17	±	6.31	±	6.96	±	5.21	±	5.32
Stride Length		0.99		1.19		1.24		1.32		1.39		1.44		1.47		1.50
[m]	±	0.20	±	0.25	±	0.22	±	0.14	±	0.15	±	0.17	±	0.11	±	0.11
(**B**)																
**CoV Gait Parameter** **± STD**	**SCA**						**ET**						**HE**			
	**Slow**		**Comf**		**Fast**		**Slow**		**Comf**		**Fast**		**Comf**		**Fast**	
CoV Trunk Horizontal RoM		0.199		0.207		0.204		0.240		0.212		0.186		0.194		0.186
	±	0.066	±	0.069	±	0.085	±	0.068	±	0.076	±	0.075	±	0.062	±	0.066
CoV Trunk Sagittal RoM		0.218		0.199		0.204		0.226		0.183		0.164		0.158		0.172
	±	0.091	±	0.061	±	0.075	±	0.070	±	0.067	±	0.066	±	0.042	±	0.051
CoV Trunk Frontal RoM		0.203		0.207		0.235		0.197		0.220		0.196		0.178		0.186
	±	0.046	±	0.042	±	0.113	±	0.070	±	0.111	±	0.088	±	0.059	±	0.063
CoV Arms RoM		0.193		0.144		0.141		0.133		0.130		0.114		0.136		0.162
	±	0.081	±	0.070	±	0.093	±	0.043	±	0.052	±	0.048	±	0.057	±	0.076
CoV Peak Arm Swing Velocity		0.142		0.109		0.111		0.129		0.114		0.097		0.120		0.118
	±	0.031	±	0.047	±	0.041	±	0.068	±	0.047	±	0.033	±	0.052	±	0.041
CoV Stride Velocity		0.101		0.069		0.066		0.048		0.029		0.025		0.028		0.034
	±	0.046	±	0.069	±	0.061	±	0.021	±	0.015	±	0.009	±	0.008	±	0.010
CoV Stride Length		0.076		0.046		0.039		0.027		0.022		0.017		0.019		0.023
	±	0.034	±	0.037	±	0.032	±	0.008	±	0.008	±	0.006	±	0.006	±	0.007

**Table 2:** (**A**) Coefficients for predictive linear regression modeling for parameters with stride velocity as independent variable. (**B**) Coefficients of linear regression modeling for CoV values of parameters with stride velocity as independent variable.

(A)									
Parameter	SCA			ET			HE		
	R^2^	*p*	Regression Statistics	R^2^	*p*	Regression Statistics	R^2^	*p*	Regression Statistics
Trunk Horizontal RoM [degrees]	0.005	0.72		0.009	0.58		0.0006	0.78	
Trunk Sagittal RoM [degrees]	0.008	0.63		0.003	0.33		0.04	0.021	F(1,132) = 5.4 y = 1.9 + 0.028x
Trunk Frontal RoM [degrees]	0.08	0.14		0.01	0.49		0.003	0.62	
Arms RoM [degrees]	0.28	0.003	F(1,28) = 10.7 y = 1.32 + 0.30x	0.36	0.0001	F(1,34) = 19.3 y = −28.6 + 0.75x	0.08	0.0007	F(1,132) = 12.1 y = −0.41 + 0.38x
Peak Arm Swing Velocity [degrees/s]	0.55	<0.0001	F(1,28) = 34.8 y = −18.9 + 3.2x	0.46	<0.0001	F(1,34) = 29.6 y = −129.9 + 4.5x	0.44	<0.0001	F(1,132) = 103 y = −197 + 5.1x
Stride Length [%stature]	0.79	<0.0001	F(1,28) = 108 y = 30.0 + 0.59x	0.57	<0.0001	F(1,34) = 45.8 y = 54.6 + 0.36x	0.39	<0.0001	F(1,132) = 83.6 y = 59.2 + 0.30x
(**B**)									
**COVs**	**SCA**			**ET**			**HE**		
	**R^2^**	***p***	**Regression Statistics**	**R^2^**	***p***	**Regression Statistics**	**R^2^**	***p***	
Trunk Horizontal RoM [degrees]	0.19	0.016	F(1,28) = 6.6 y = 0.30 − 0.0015x	0.18	0.010	F(1,34) = 7.4 y = 0.37 − 0.0022x	0.003	0.555	
Trunk Sagittal RoM [degrees]	0.26	0.004	F(1,28) = 10.0 y = 0.32 − 0.0018x	0.17	0.014	F(1,34) = 6.8 y = 0.34 − 0.002x	0.02	0.129	
Trunk Frontal RoM [degrees]	0.12	0.065		0.005	0.69		0.001	0.71	
Arms RoM [degrees]	0.14	0.04	F(1,28) = 4.5 y = 0.25 − 0.0015x	0.13	0.028	F(1,34) = 5.3 y = 0.22 − 0.0012x	0.03	0.55	
Peak Arm Swing Velocity [degrees/s]	0.46	<0.0001	F(1,28) = 23.9 y = 0.20 − 0.0013x	0.09	0.084		0.00001	0.86	
Stride Length [%stature]	0.64	<0.0001	F(1,28) = 50.7 y = 0.14 − 0.0014x	0.33	0.00013	F(1,34) = 18.6 y = 0.05 − 0.00033x	0.00001	0.99	

## Data Availability

The clinical data and gait assessments are not publicly available due to federal and institutional data privacy regulations of patient data and the General Data Protection Regulation of the European Union. Patient consent for public sharing of their data was not obtained, but the processed data can be made available from the corresponding author upon reasonable request in an anonymized manner in the framework of a data sharing agreement.

## Supplementary Materials

The following supporting information can be downloaded at: https://www.mdpi.com/article/10.3390/s24113476/s1. Table S1: Clinical characteristics of SCA cohort; Figure S1: Walking speed categories for patients with SCA; Figure S2: Walking speed categories for patients with ET; Figure S3A,B: Regression modelling of parameter dependence of gait velocity in ET; Figure S4: Walking speed categories for HE; Figure S5 A&B: Regression modelling of parameter dependence of gait velocity in HE; Figure S6: Cohort differences per walking speed category.
