# Supplementary material for: Gait Variability as a Potential Motor Marker of Cerebellar Disease—Relationship between Variability of Stride, Arm Swing and Trunk Movements, and Walking Speed
Source: Sensors (Basel). 2024 May 28;24(11):3476. doi: 10.3390/s24113476 (PMC11174553; doi:10.3390/s24113476)

Supp. Table S1 – Clinical characteristics of patient cohort

ID	Age at assessment	Genetic findings	Sex	Height	Weight	SARA total	SARA Gait	SARA Stance	SARA Sitting
P1	71	c368G>C, pG123A heterozygot	f	181	74	11	2	2	1
P2	69	c244-252delACCTTCGAG heterozygot	m	175	74	14,5	2	2	1
P3	32	c197G>A, pC66Y (Exon2) (son of P4)	m	171	63	9,5	2	0	0
P4	61	c197G>A, pC66Y (Exon2)	f	157	74,5	20,5	3	4	2
P5	64	c197G>A, pC66Y (Exon2) (sister of P4)	f	147	na	9,5	2	2	0
P6	62	brother of P2, not tested	m	177	89	11	2	2	0
P7	53	c.353G>A p.E11BD (PRKCG) heterozygot	m	175	85	9.5	1	2	0
P8	58	c.338_340delTCT, p.F113_C114delinsC (Exon 4)	f	167	93	9,5	3	2	0
P9	45	c.392G>C, p.C131S	f	159	78	9,5	2	1	0
P10	27	c.392G>C, p.C131S (son of P19)	m	173	120	3	0	0	0
**average**	**54.2**		**5f, 5m**	**168.2**	**83.4**	**10.7**	**1.9**	**1.7**	**0.4**
**median**	**59.5**			**172**	**78**	**9.5**	**2**	**2**	**0**

Supplemental Figure S1 – Walking speed categories for patients with SCA

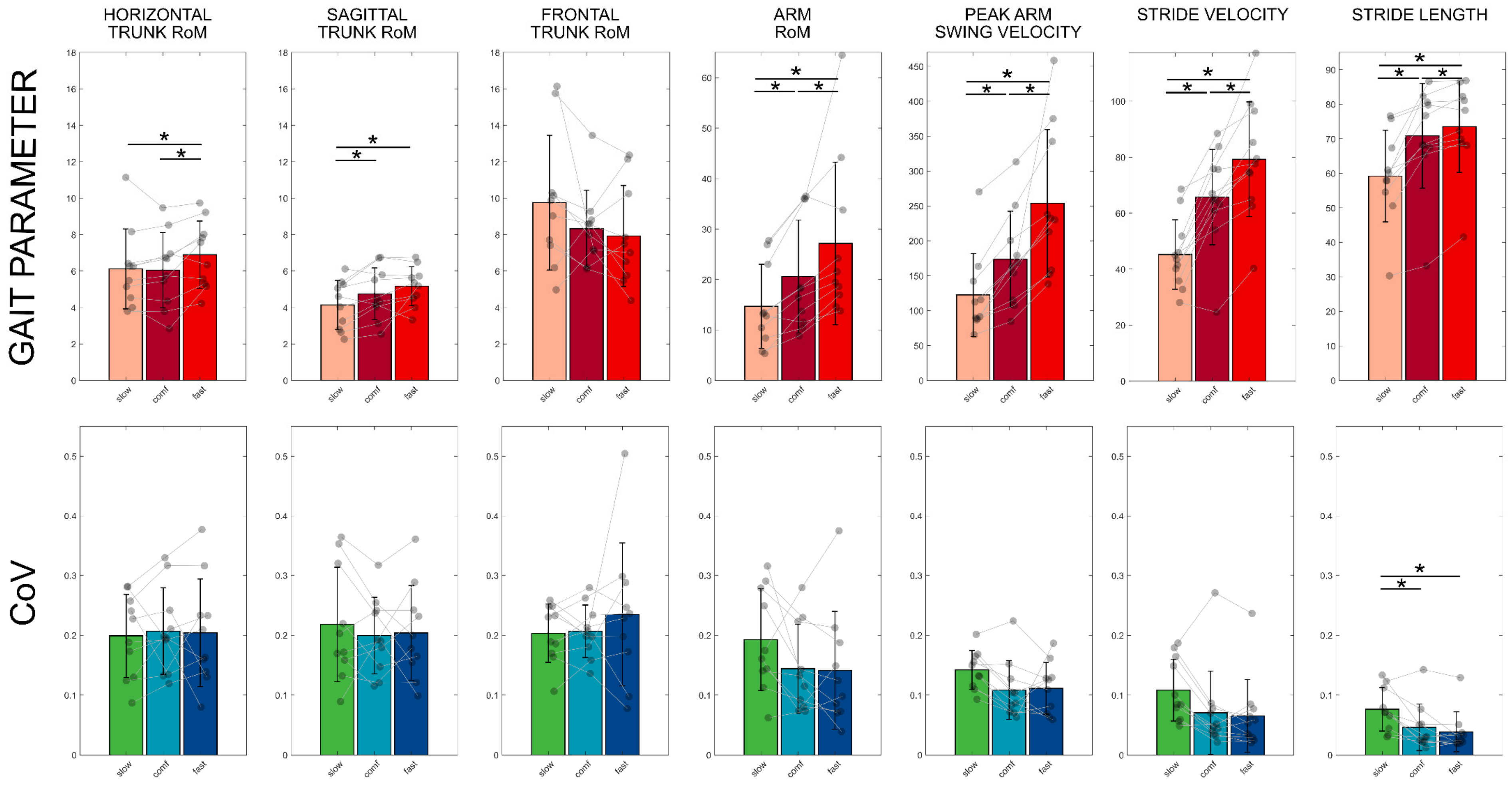

Supplemental Figure S2 – Walking speed categories for patients with ET

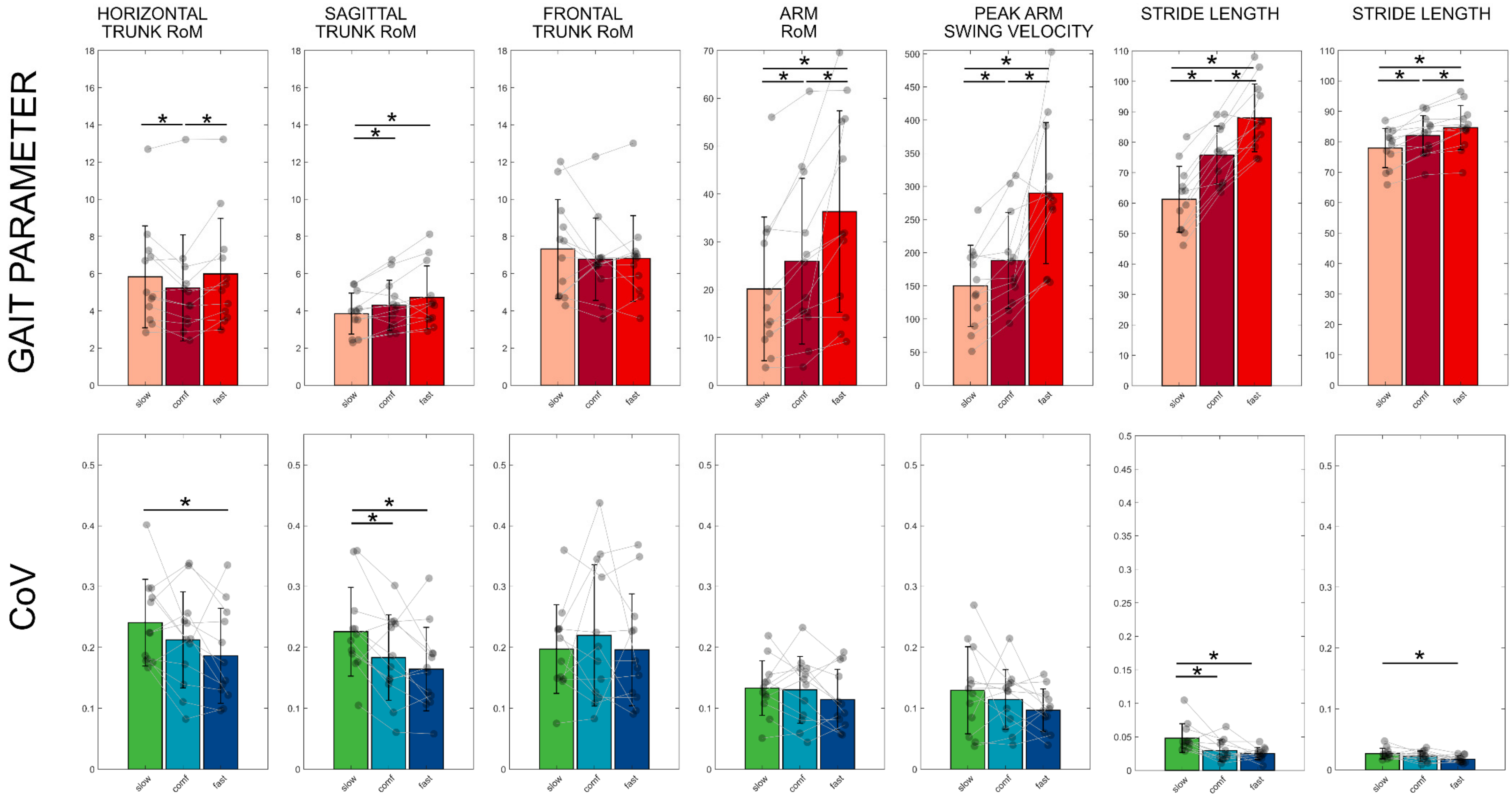

Supplemental Figure S3A & B Regression modelling of parameter dependence of gait velocity in ET

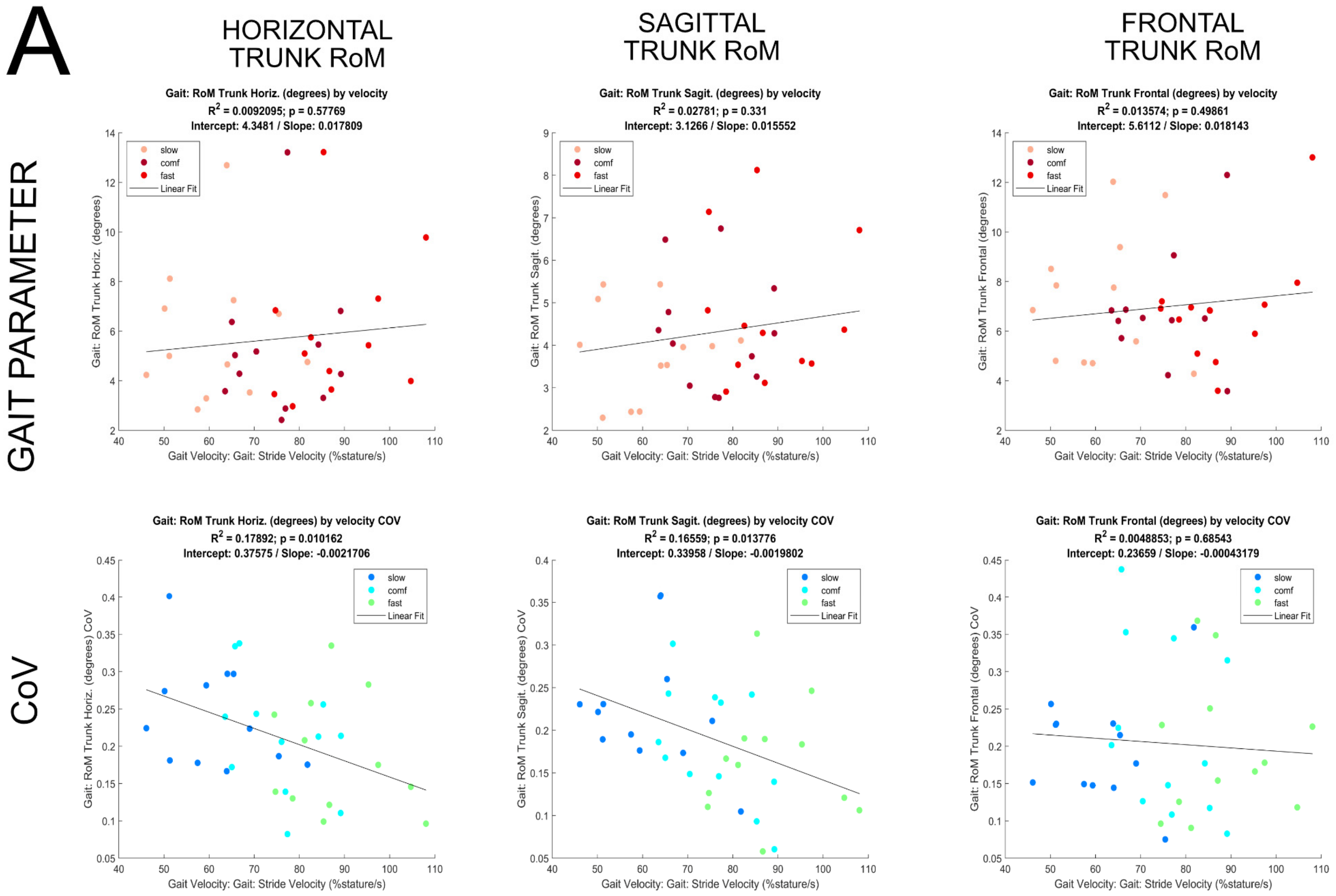

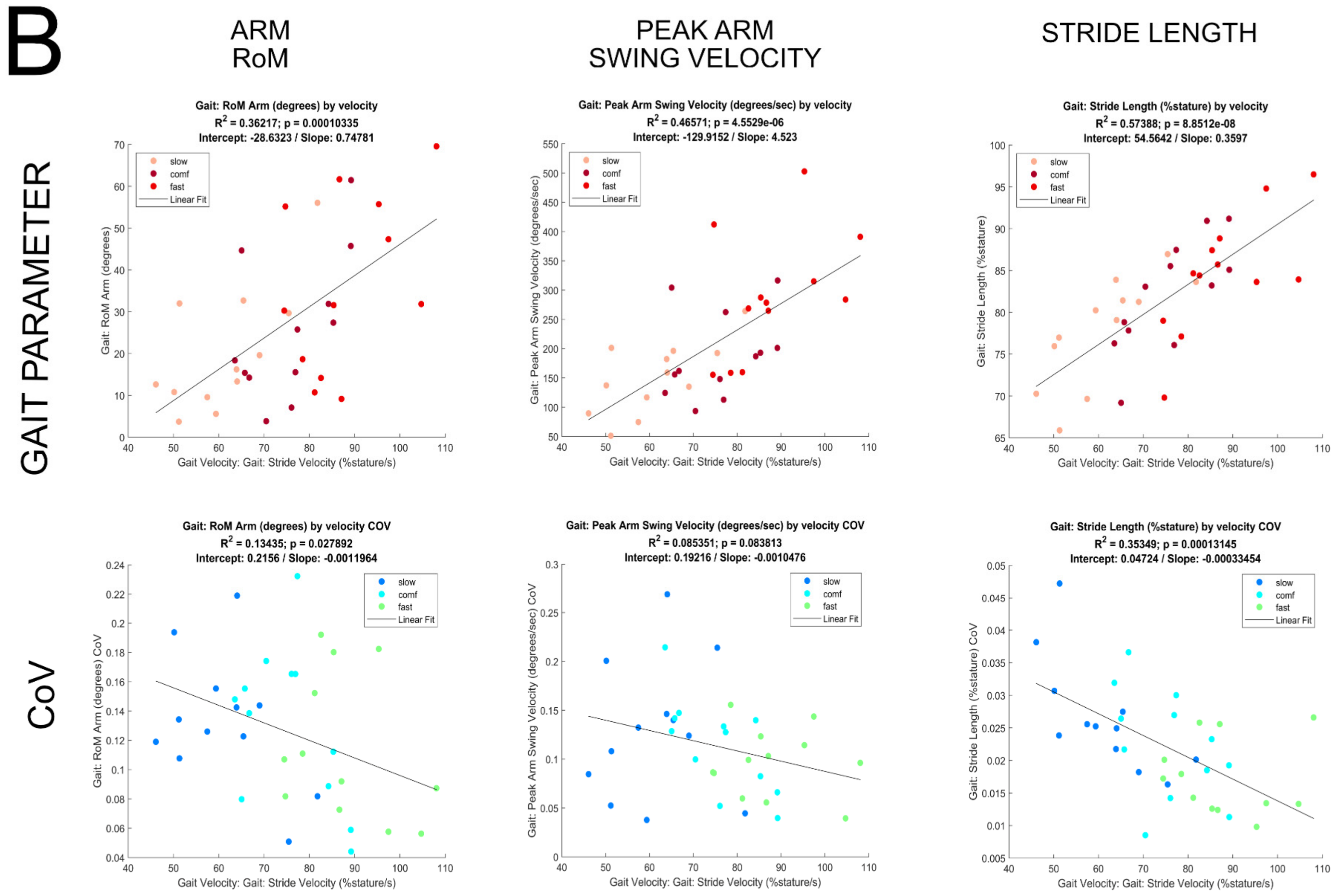
B
ARM RoM
PEAK ARM SWING VELOCITY
STRIDE LENGTH
GAIT PARAMETER
CoV
Gait: RoM Arm (degrees) by velocity
R2 = 0.36217; p = 0.00010335
Intercept: -28.6323 / Slope: 0.74781
Gait: RoM Arm (degrees)
Gait: Peak Arm Swing Velocity (degrees/sec) by velocity
R2 = 0.46571; p = 4.5529e-06
Intercept: -129.9152 / Slope: 4.523
Gait: Peak Arm Swing Velocity (degrees/sec)
Gait: Stride Length (%stature) by velocity
R2 = 0.57388; p = 8.8512e-08
Intercept: 54.5642 / Slope: 0.3597
Gait: Stride Length (%stature)
slow
comf
fast
Linear Fit
Gait Velocity: Gait: Stride Velocity (%stature/s)
Gait: RoM Arm (degrees) by velocity COV
R2 = 0.13435; p = 0.027892
Intercept: 0.2156 / Slope: -0.0011964
Gait: RoM Arm (degrees) CoV
Gait: Peak Arm Swing Velocity (degrees/sec) by velocity COV
R2 = 0.085351; p = 0.083813
Intercept: 0.19216 / Slope: -0.0010476
Gait: Peak Arm Swing Velocity (degrees/sec) CoV
Gait: Stride Length (%stature) by velocity COV
R2 = 0.35349; p = 0.00013145
Intercept: 0.04724 / Slope: -0.00033454
Gait: Stride Length (%stature) CoV

Supplemental Figure S4 – Walking speed categories for patients with HE

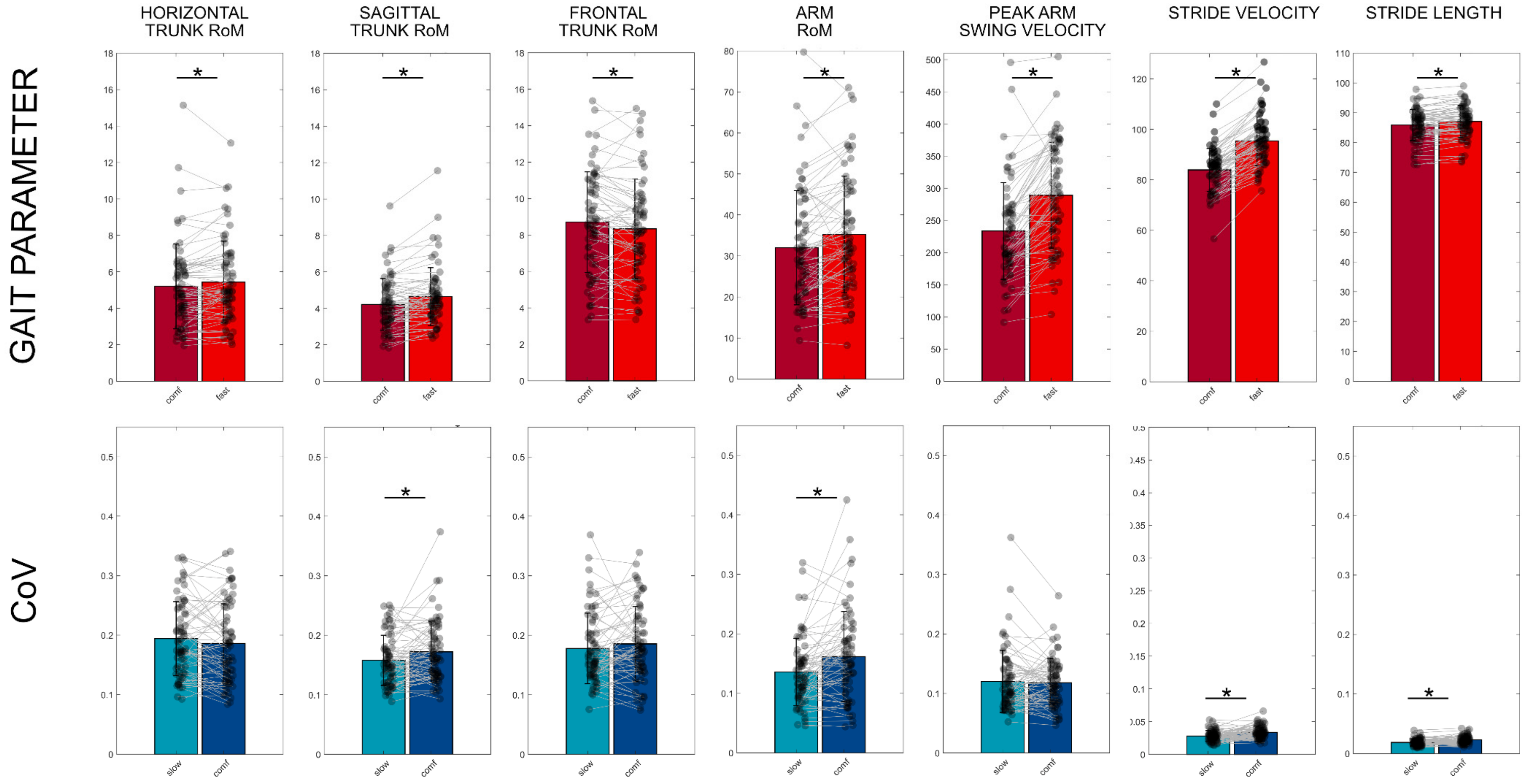

Supplemental Figure S5A &B Regression modelling of parameter dependence of gait velocity in HE

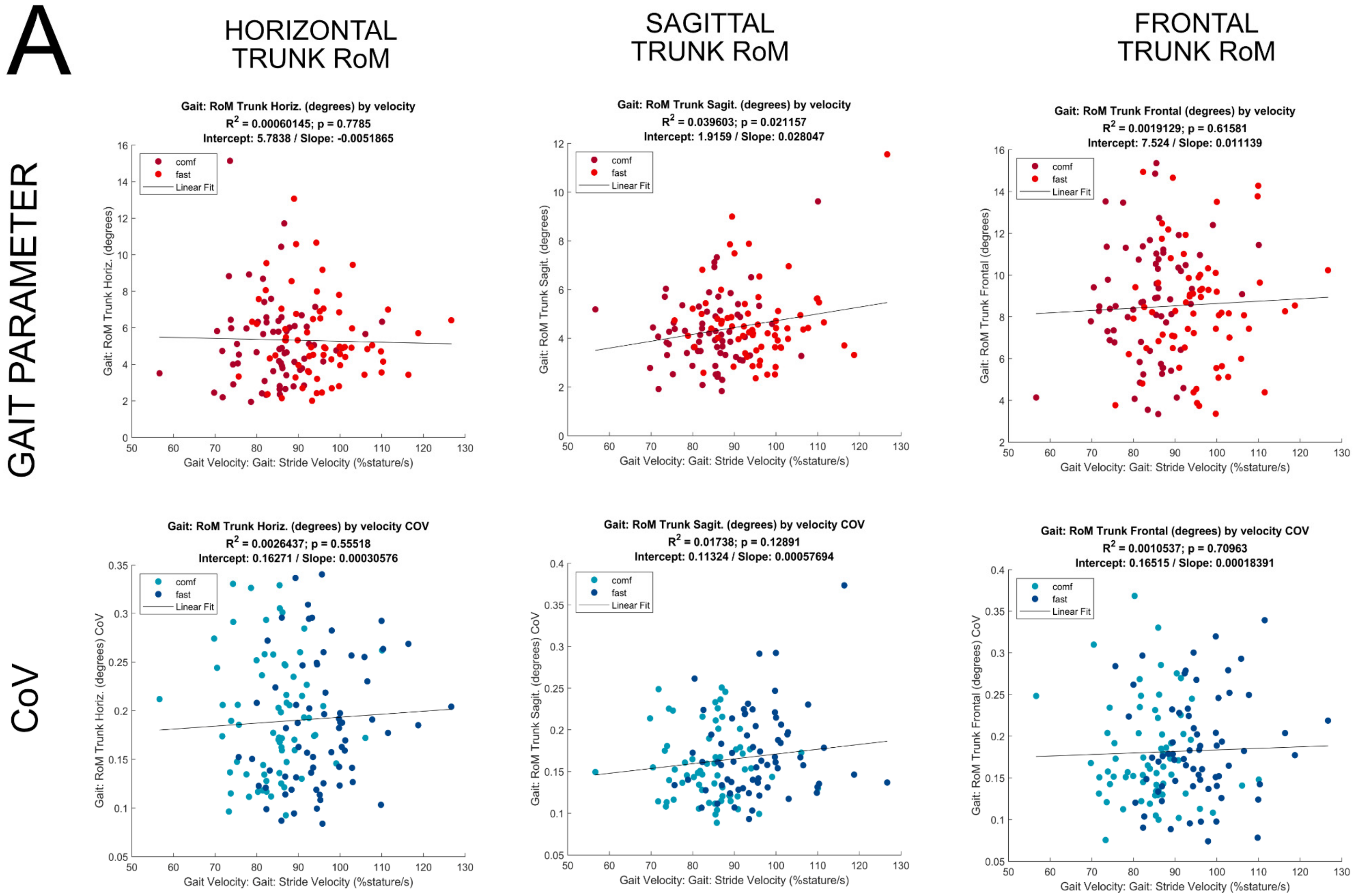

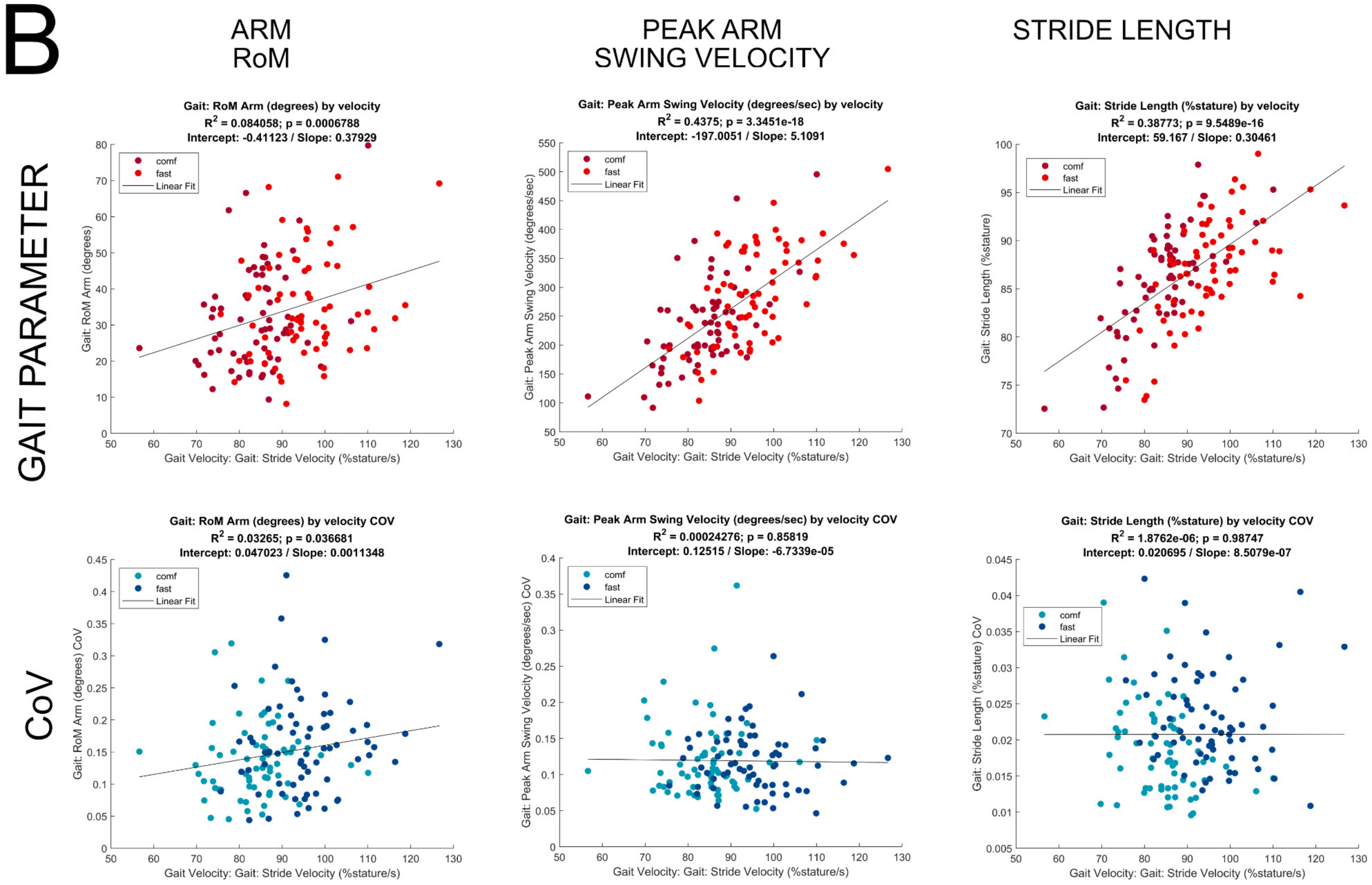
B
ARM RoM
PEAK ARM SWING VELOCITY
STRIDE LENGTH
GAIT PARAMETER
CoV
Gait: RoM Arm (degrees) by velocity
R^2 = 0.084058; p = 0.0006788
Intercept: -0.41123 / Slope: 0.37929
Gait: Peak Arm Swing Velocity (degrees/sec) by velocity
R^2 = 0.4375; p = 3.3451e-18
Intercept: -197.0051 / Slope: 5.1091
Gait: Stride Length (%stature) by velocity
R^2 = 0.38773; p = 9.5489e-16
Intercept: 59.167 / Slope: 0.30461
Gait: RoM Arm (degrees) by velocity COV
R^2 = 0.03265; p = 0.036681
Intercept: 0.047023 / Slope: 0.0011348
Gait: Peak Arm Swing Velocity (degrees/sec) by velocity COV
R^2 = 0.00024276; p = 0.85819
Intercept: 0.12515 / Slope: -6.7339e-05
Gait: Stride Length (%stature) by velocity COV
R^2 = 1.8762e-06; p = 0.98747
Intercept: 0.020695 / Slope: 8.5079e-07
comf
fast
Linear Fit
Gait: RoM Arm (degrees)
Gait: Peak Arm Swing Velocity (degrees/sec)
Gait: Stride Length (%stature)
Gait: RoM Arm (degrees) CoV
Gait: Peak Arm Swing Velocity (degrees/sec) CoV
Gait: Stride Length (%stature) CoV
Gait Velocity: Gait: Stride Velocity (%stature/s)

Figure S6 – Cohort differences per walking speed category

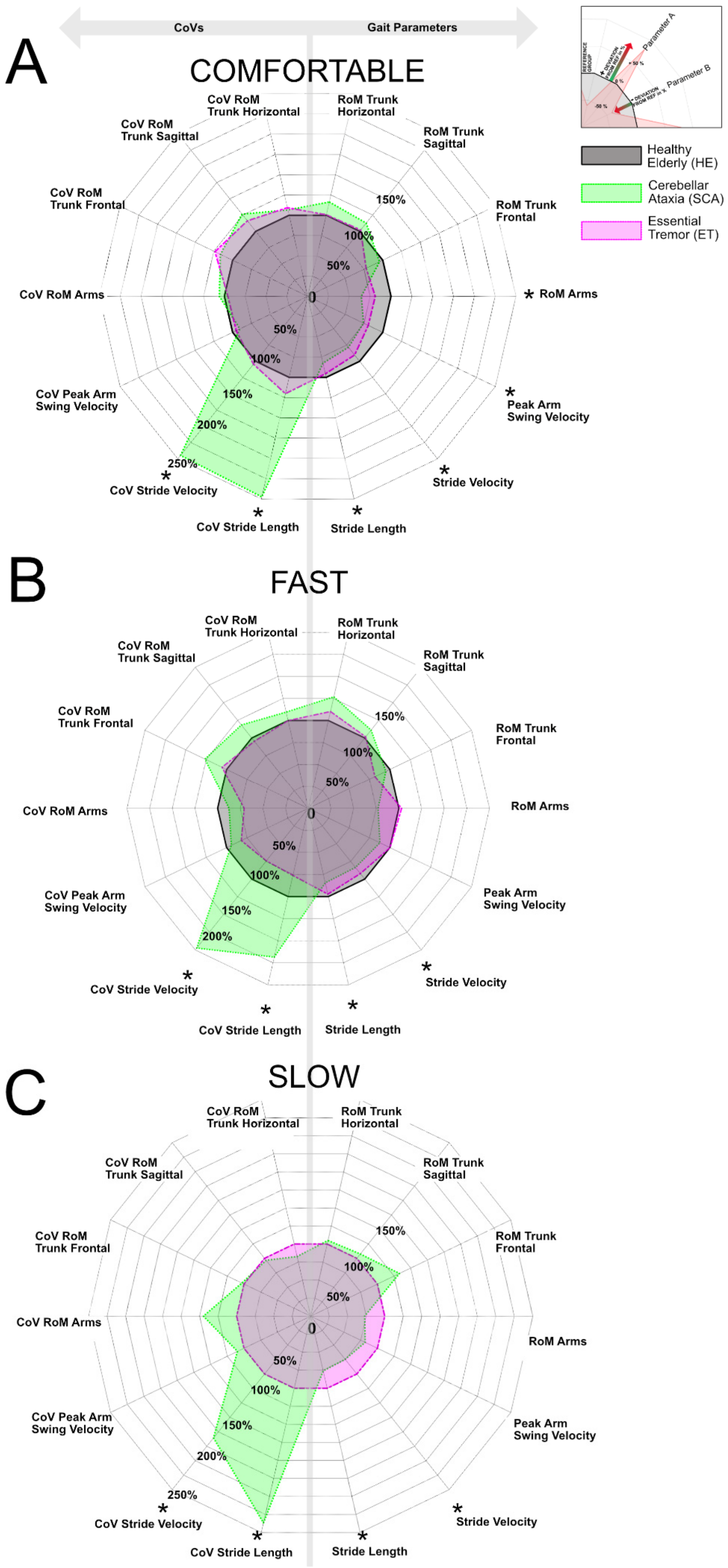

Supplement: Supplementary file 1 [file sensors-24-03476-s001.zip › sensors-2986923-supplementary.pdf]
